# miRNA Temporal Analyzer (mirnaTA): a bioinformatics tool for identifying differentially expressed microRNAs in temporal studies using normal quantile transformation

**DOI:** 10.1186/2047-217X-3-20

**Published:** 2014-10-13

**Authors:** Regina Z Cer, J Enrique Herrera-Galeano, Joseph J Anderson, Kimberly A Bishop-Lilly, Vishwesh P Mokashi

**Affiliations:** 1 Biological Defense Research Directorate, Naval Medical Research Center-Frederick, 8400 Research Plaza, Fort Detrick, MD 21702, USA; 2 Henry M. Jackson Foundation for the Advancement of Military Medicine, 6720-A Rockledge Drive, Suite 100, Bethesda, MD 20817, USA; 3 Chem Bio Research Center of Excellence, Defense Threat Reduction Agency, 2800 Bush River Road E2800-b198, Aberdeen Proving Ground, MD 21010, USA

**Keywords:** microRNA, miRNA Temporal Analyzer, mirnaTA, Time series, Differential expression, DE, Quantile normalization, Linear model, Normal quantile transformation

## Abstract

**Background:**

Understanding the biological roles of microRNAs (miRNAs) is a an active area of research that has produced a surge of publications in PubMed, particularly in cancer research. Along with this increasing interest, many open-source bioinformatics tools to identify existing and/or discover novel miRNAs in next-generation sequencing (NGS) reads become available. While miRNA identification and discovery tools are significantly improved, the development of miRNA differential expression analysis tools, especially in temporal studies, remains substantially challenging. Further, the installation of currently available software is non-trivial and steps of testing with example datasets, trying with one’s own dataset, and interpreting the results require notable expertise and time. Subsequently, there is a strong need for a tool that allows scientists to normalize raw data, perform statistical analyses, and provide intuitive results without having to invest significant efforts.

**Findings:**

We have developed miRNA Temporal Analyzer (mirnaTA), a bioinformatics package to identify differentially expressed miRNAs in temporal studies. mirnaTA is written in Perl and R (Version 2.13.0 or later) and can be run across multiple platforms, such as Linux, Mac and Windows. In the current version, mirnaTA requires users to provide a simple, tab-delimited, matrix file containing miRNA name and count data from a minimum of two to a maximum of 20 time points and three replicates. To recalibrate data and remove technical variability, raw data is normalized using Normal Quantile Transformation (NQT), and linear regression model is used to locate any miRNAs which are differentially expressed in a linear pattern. Subsequently, remaining miRNAs which do not fit a linear model are further analyzed in two different non-linear methods 1) cumulative distribution function (CDF) or 2) analysis of variances (ANOVA). After both linear and non-linear analyses are completed, statistically significant miRNAs (P < 0.05) are plotted as heat maps using hierarchical cluster analysis and Euclidean distance matrix computation methods.

**Conclusions:**

mirnaTA is an open-source, bioinformatics tool to aid scientists in identifying differentially expressed miRNAs which could be further mined for biological significance. It is expected to provide researchers with a means of interpreting raw data to statistical summaries in a fast and intuitive manner.

## Findings

MicroRNAs (miRNAs) are short single-stranded non-coding RNAs approximately 19–22 nucleotide long, which are critical regulators of gene expression and have been implicated in a wide range of physiological processes, such as apoptosis and growth, as well as pathological processes, including inflammatory responses, cancer, neurodegenerative and cardiovascular diseases [1-7]. This rapid growth is evident by the exponentially increasing number of miRNAs reported in the recent Release 21 (June 2014) of miRBase [8,9] which contains 35,828 mature miRNA products in 223 different species. Accompanying this growth is the development of many miRNA discovery bioinformatics tools including, but not limited to miRscan [10], miRFinder [11], miRDeep [12] and miRanalyzer [13], to help researchers identify miRNAs from existing miRNA databases and/or predict novel miRNAs from NGS data.

In recent years, the number of miRNA experiments involving multiple time-series has largely increased [14]. This is not surprising since biological processes are often dynamic, and therefore, a cross-sectional study based on a single time point would not provide important information that time-course studies can provide. Particularly in miRNA studies, researchers often implement multiple-time points to capture how certain miRNAs may display transient expression changes over the course of treatment or infection over time. For instance, Jayaswal *et al.* carried out a drug study involving a multiple myeloma cell line, U266, and consisting of six time points—0, 2, 4, 8, 24, and 48 hours with two biological replicates per time point for both miRNA and mRNA [15]. In another study by Li Z *et al.*, miRNA expression profiles were assessed in the mouse livers in a time-course experiment at days 1, 3, 7, 15, 30 and 120 post treatment [16].

Despite the fact that there are several publications dealing with the statistical analysis of time-series expression data, there are significant computational challenges in making sense of the massive and complex datasets produced by time-series experiments. There are a number of differential expression (DE) tool packages including STEM [17], MaTSE [18], Linear Models for Microarray Data (LIMMA) [19], Significance Analysis of Microarrays (SAM) [20], Extraction of Differential Gene Expression (EDGE) [21], and Bayesian Estimation of Temporal Regulation (BETR) [22]. In miRNA research in particular, the two most commonly used DE tools are edgeR [23] and DEseq [24]. However, learning to use these tools requires the expertise of bioinformaticians as they often come with several package dependencies and not every research laboratory employs these specialists nor has advanced computing infrastructure. There is a crucial need for a simple tool that would allow any bench scientist to analyze the differential expression of miRNAs or other subjects in temporal studies.

### Development of mirnaTA

**Figure 1:**
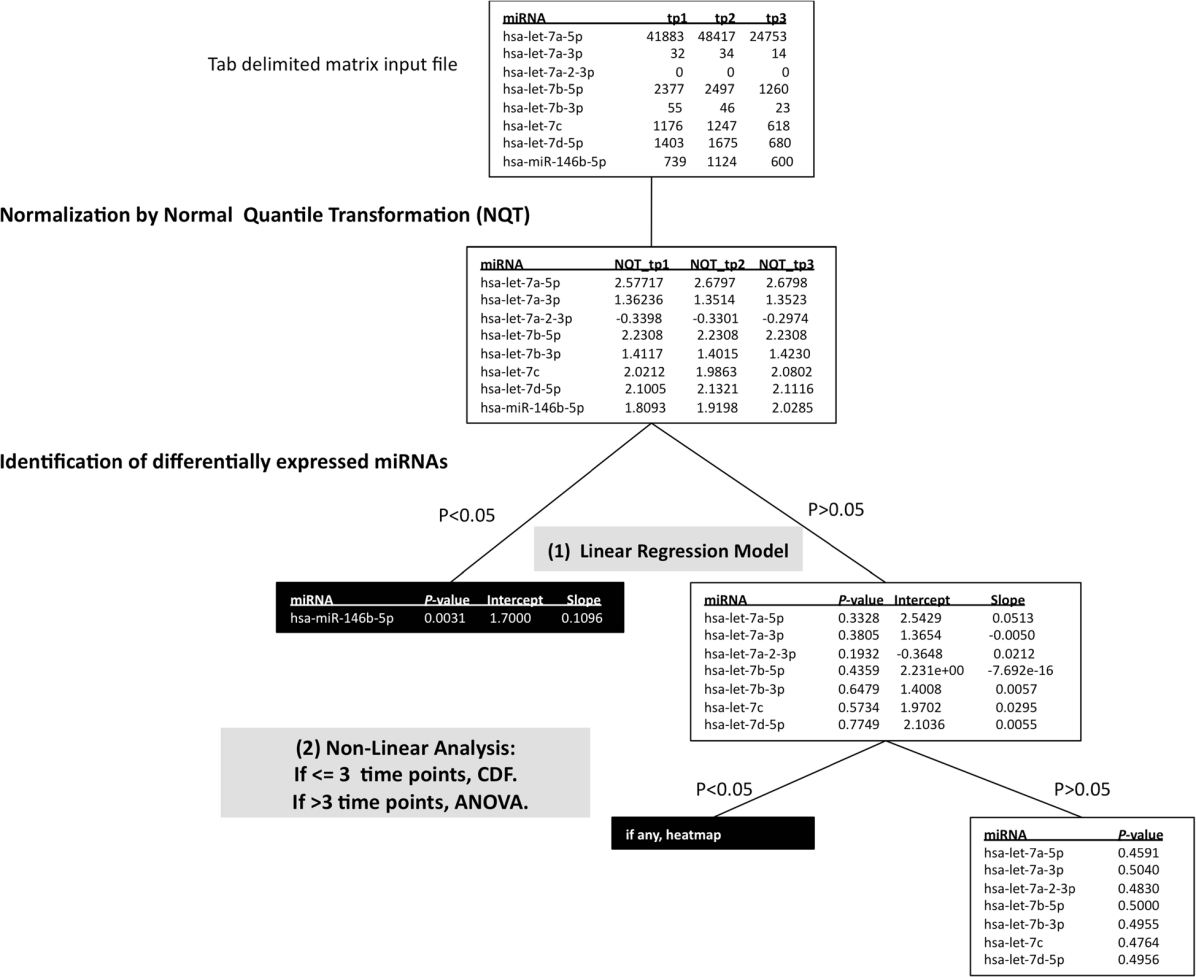
**An overview of the mirnaTA workflow using a three time point example.** mirnaTA converts the tab-delimited, matrix raw data into quantiles using Normal Quantile Transformation (NQT). Then, using a linear regression model, mirnaTA identifies miRNA species which either increase or decrease linearly. Any miRNA species with *P* < 0.05 are considered to be statistically significant (shown in the black box). miRNAs that did not fit into a linear model with statistical significance are further analyzed using either cumulative distribution function (CDF) or analysis of variance (ANOVA) depending on the number of time points.

The miRNA Temporal Analyzer (mirnaTA) is a by-product of a recent study where human peripheral blood mononuclear cells (hPBMCs) were exposed to bacterial pathogens and assayed at three different time points. Briefly, total RNA was extracted, miRNA sequencing libraries were constructed, sequencing was performed on the MiSeq sequencer, and miRNA sequences were identified from sequencing reads using a standard procedure (see in Additional file 1: Figure S1). Then, mirnaTA was used to identify differentially expressed miRNAs for further validation in the laboratory [Cer *et al.*, unpublished data]. mirnaTA performs a number of distinct steps (Figure 1): (i) normalization of the raw count data into quantiles using Normal Quantile Transformation (NQT), (ii) analysis of NQT data to locate any miRNA species which are either increasing or decreasing linearly using linear regression model, (iii) further analysis of miRNA species that did not fit in linear model by two different methods: (a) normal distribution function known as cumulative distribution function (CDF) if the number of time points is = < 3 or (b) analysis of variances (ANOVA) if the number of time points is >3, (iv) generation of heat maps for any miRNAs that are differentially expressed with statistical significance (P < 0.05), and (v) providing intuitive HTML output formats. Some of these steps are explored in more detail below.

#### *Input file requirement*

Any datasets resulting from RNA-seq experiments, array-based miRNA profiling or RT-PCR experiments, can be used as input data for the mirnaTA as long as the tab-delimited input files contain two columns, miRNA name and sequence read count of the miRNA. The number of time points that can be submitted to mirnaTA is a minimum of 2 to a maximum of 20. mirnaTA is able to handle to three replicates as follows: if the correlation coefficient (r) value between two replicates is more than or equal to 0.70, the count values from replicates are averaged and used in the study. Incremental correlation is applied for each additional replicate (see User Guide available at [25] for more details). If the r value is less than 0.7, the replicate data is not used in the calculations. The correlation coefficient, r value 0.7 was chosen arbitrarily as it was deemed reasonable for replicates to be considered sufficiently correlated. However, users are able to change this to any value although we do not recommend to use any value below 0.7.

#### *Data normalization by NQT*

Data normalization, a critical step of DE analysis, attempts to remove technical variability while preserving biological significance. Although there is no consensus on the best method for the normalization of microRNA sequencing data, when seven commonly used normalization methods, namely, global normalization, Lowess normalization, trimmed mean method (TMM), quantile normalization, scaling normalization, variance stabilization (VSN) and invariant method (IN), were evaluated, the Lowess normalization and the quantile normalization were found to be the best approaches [26]. It should be noted that edgeR uses TMM and DEseq uses a negative binomial (NB) model. mirnaTA is different in that it implements quantile normalization (also referred here as (NQT)) chosen for its robustness and reduced bias. Quantile normalization is a rank-based procedure which scales data within each quantile separately and has been shown to have bias near zero relative to qRT–PCR [27]. Another factor considered in choosing quantile-based scaling is its medium computational complexity [28]. Briefly, raw data is normalized using NQT as follows: random numbers are drawn from the normal distribution based on the number of observations (here the number of miRNA species) and these numbers are sorted (Equation 1). Then quantiles are assigned according to each rank (Equations 2–3). All the equations included here are R language commands unless or otherwise specified:

(1) $$rn\;=\;\text{sort}\left(\text{rnorm}\left(\text{no}\_\text{of}\_\text{miRNAs}\right)\right)$$

(2) $$\text{qt}\;=\;\text{rank}\left(x\right)/\text{length}\left(x\right)$$

(3) $$nqt\;=\;rn\left[\text{rank}\left(\text{qt}\right)\right]$$

#### *Identification of differentially expressed miRNAs by a linear regression model*

Using a linear regression, quantile transformed data is analyzed to identify any miRNA species which display either increasing or decreasing expression. Using NQT data over different time points, the corresponding *P*-values, intercept, and slope values are calculated (Equations 4–8), and any miRNAs with P <0.05 are considered to be statistically significant:

(4) $$\text{lmod}\;=\;\text{lm}\;\left(y\;∼\;x\right)\;\text{where}\;x\;=\;\text{time},y\;=\;NQT\;\text{data}$$

(5) $$s\;=\;\text{summary}\left(\text{lmod}\right)$$

(6) $$\begin{array}{c} p=as.\text{character}\left(pf\left(\text{s\$fstatistic}\left[1\right],\text{s\$fstatistic}\left[2\right],\right)\right)\\ \;\left(s\$\text{fstatistic}\left[3\right],\text{lower}.\text{tail}\;=\;\text{FALSE}\right) \end{array}$$

(7) $$\text{intercept}\;=\;s\$\text{coefficients}\left[1\right]$$

(8) $$\text{slope}\;=\;\text{s\$coefficients}\left[2\right]$$

#### *Identification of other differentially expressed miRNA species using a non-linear analysis*

The remaining miRNA species that did not fit in a linear model are further analyzed depending on the number of time points in the experiment: (a) the cumulative distribution function (CDF) is applied when the number of time points is = <3 (Equation 9), and (b) without the assumption of equal variances for all groups, the analysis of variance (ANOVA) [29] is applied when the number of time points is >3. Similar to linear analysis criteria, any miRNAs with P <0.05 are considered to be statistically significant.

(9) $$\begin{array}{c} cdf\_\text{pval}\;=\;1-\text{pnorm}((-as.\text{numeric}\left(tpn\right)\\ \;-as.\text{numeric}\left(tp1\right),\text{mean}\;=\;0,\;sd\\ \;=\;1,\;\text{lower}.\text{tail}\;=\;\text{TRUE},\;\text{log}.p\;\\ \;=\;\text{FALSE}))\;\text{where}\;tp1\\ \begin{array}{l} \;=\;nqt\;\text{data}\;for\;\text{time}\;\text{point}\;1\;\text{and}tpn\\ \;=nqt\;\text{data}\;for\;\text{time}\;\text{point}\;2\;\text{or}\;\text{time}\;\text{point}\;3 \end{array} \end{array}$$

(10) $$\begin{array}{l} \text{anova}\_\text{pval}\;=\;\text{oneway}.\text{test}\left(y∼x\right)\text{where}\;y\;=\;nqt\;\text{data}\;\text{and}\;x\;=\\ \text{time}\;\text{points}\;\text{divided}\;\text{into}\;\text{two}\;\text{groups} \end{array}$$

#### *Syntax for running mirnaTA*

The syntax to run mirnaTA is simple, as follows: *perl nmrc_mirnata.pl -i < input_file > -n < x > -t < y>*, where nmrc_mirnata.pl is the wrapper Perl script, < input_file > is a tab-delimited, matrix file, x = the number of replicates, y = the number of time points. For example, to run mirnaTA for a dataset with three time points, two replicates and correlation coefficient value of 0.85 (default is 0.7), the syntax would be “*perl nmrc_mirnata.pl -i example_datasets/3tp2rep_dataset -n 2 –t 3 –c 0.85*”. To demonstrate the functionality of mirnaTA, a comprehensive User Guide with examples using different datasets and expected results are provided at our SourceForge [25] page and also archived in GigaDB [30].

#### *Visualizations of mirnaTA outputs*

**Figure 2:**
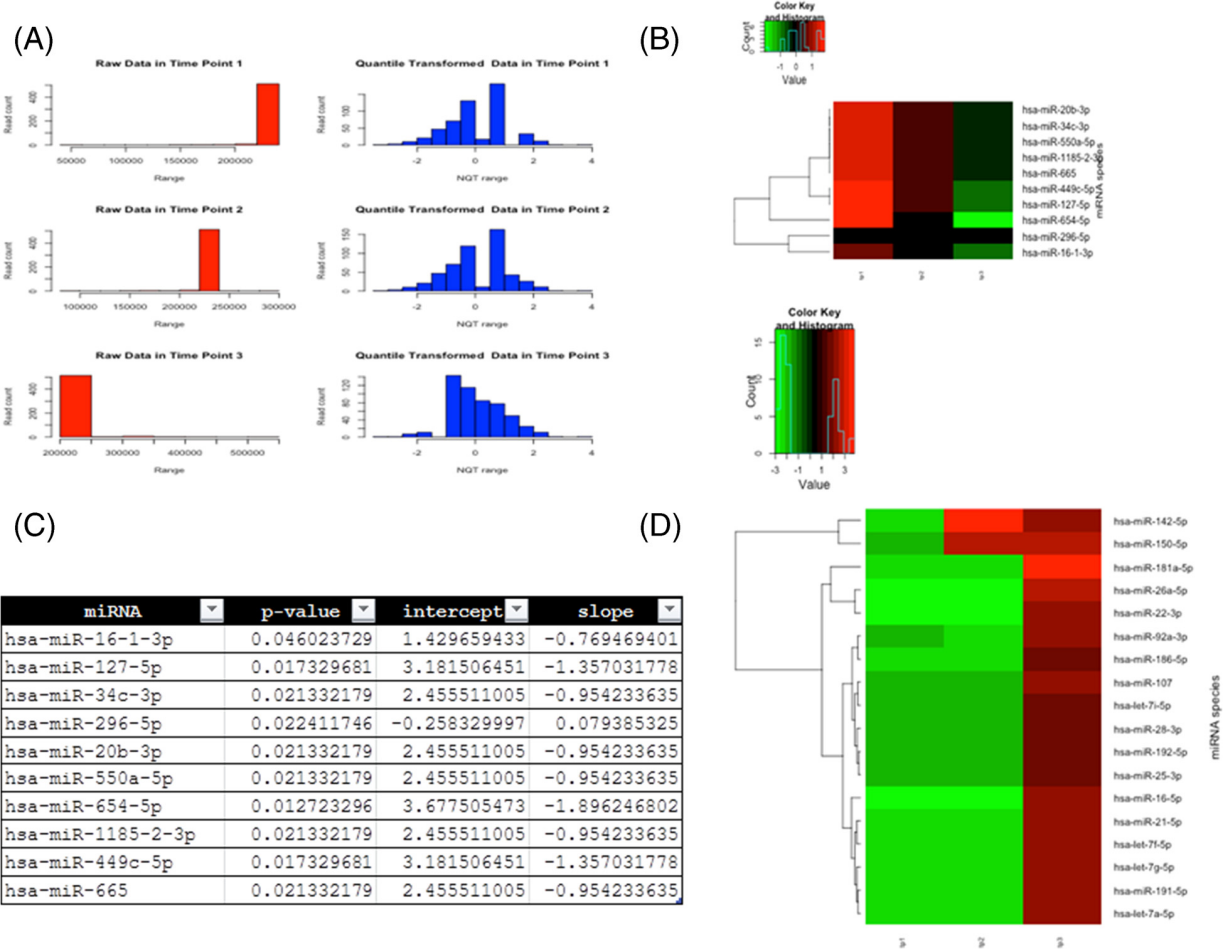
**mirnaTA Outputs. (A)** A Portable Network Graphic (PNG) image showing the raw data (before normalization) vs. NQT data (after normalization). **(B)** A heat map of differentially expressed miRNAs with statistical significance (P < 0.05) which were identified to be linearly increasing or decreasing using linear regression model. **(C)** One of the many intermediate data files (shown here is a text file of P-values, intercept and slope data of significant miRNAs). **(D)** A heat map of differentially expressed miRNAs with statistical significance (P < 0.05) which were identified to be increasing or decreasing using either cumulative distribution function (CDF) of the normal distribution or analysis of variance (ANOVA). Note that all these images and text files are available for viewing on web browsers by opening ‘mirnata.html’ file in ‘output_files’ directory.

mirnaTA provides a rich source of HTML format data, including raw data, quantile transformed data, a heat map of differentially expressed miRNAs (P < 0.05) which were identified to be linearly increasing or decreasing using a linear regression model, as well as a heat map of differentially expressed miRNAs (P < 0.05) which were identified to be increasing or decreasing using either a cumulative distribution function (CDF) or an analysis of variance (ANOVA), if present. Also included are several intermediate files including a list of significant miRNA species with their *P*-values, slope, and intercept values (Figure 2). All these information is integrated into an HTML interface which can be viewed in any web browser. It should be noted that the heat map text files may also be visualized in other software packages such as PermutMatrix [31].

## Conclusions

mirnaTA is an open-source bioinformatics tool that can be run in Linux, Mac or Windows with Perl and R package dependencies. While there are many other time-series analysis tools available, the main advantages of mirnaTA are:

(1). Simplicity: Even though it is a command line program and users need to be aware of corrections for multiple testing, they need to provide only one input file and enter one line of Perl syntax, and do not need to have a strong statistical or computational background.

(2). Reliability: The statistically significant miRNA species identified by linear regression model have met very stringent criteria, and therefore could be further examined for confirmation or validation in wet laboratories.

(3). Practicality: The mirnaTA only requires a simple input file which can be prepared by anyone, takes up to three replicates and 20 time points, and generates publication quality graphics in PNG format.

(4). Wide applicability: The mirnaTA can be used to analyze any kind of data (gene, weather data, weight, etc.), and does not have to be restricted to miRNA data. All a user has to do is to replace the miRNA name in the first column with another subject of study.

In summary, although mirnaTA does not yet provide researchers with “a push-button” solution for differential expression analysis, it gives them the power to use a simple Perl syntax to not only identify differentially expressed miRNAs, but also automatically generate intuitive graphical outputs. This simple and automatic implementation of previously detached concepts in mirnaTA could be instrumental in saving a significant amount of time for many basic research scientists.

## Availability and requirements

**Project name**: miRNA Temporal Analyzer (mirnaTA)

**Project home page**: http://sourceforge.net/projects/mirnata

**Operating system (s)**: Linux, Mac and Windows

**Programming languag**e: Perl and R

**Other Requirements**: None

**License**: The GNU Lesser General Public License, version 3.0 (LGPL-3.0)

**Any restrictions to use by non-academics**: None

## Availability of supporting data

Archival version of the supporting files, user guide and additional datasets used in the paper are hosted in the *GigaScience* GigaDB database [30], and for the most up to date versions please see the source forge page: http://sourceforge.net/projects/mirnata.

## Abbreviations

ANOVA: Analysis of variance; CDF: Cumulative distribution function; DE: Differential expression; miRNA: microRNA; NGS: Next-generation sequencing; NQT: Normal quantile transformation; PNG: Portable network graphics; TMM: Trimmed mean method.

## Competing interests

The authors declare that they have no competing interests.

## Authors’ contributions

RZC wrote Perl and R scripts, packaged the workflow, released code and prepared the manuscript. JEH wrote custom R functions and oversaw R statistical analyses. JJA tested the package and provided patches. KAB tested the package and edited the manuscript. VPM oversaw the project and gave scientific advice. All authors read, contributed and approved the final manuscript.

## Supplementary Material

Additional file 1: Figure S1 Detailed steps for generating input files for mirnaTA. FASTQ files generated from any NGS sequencing platform are converted into FASTA files. Artificially introduced 3′ adapter sequences are trimmed, and post-trimmed reads that are a minimum of 15 base pairs are filtered against contaminants. Reads that do not match to contaminants are screened for mature miRNA species (black box) which are further analyzed for statistical significance using mirnaTA. Click here for file
