# Supplementary material for: miRNA Temporal Analyzer (mirnaTA): a bioinformatics tool for identifying differentially expressed microRNAs in temporal studies using normal quantile transformation
Source: Gigascience. 2014 Oct 13;3:20. doi: 10.1186/2047-217X-3-20 (PMC4212236; doi:10.1186/2047-217X-3-20)
Supplement: Additional file 1: Figure S1 — Detailed steps for generating input files for mirnaTA. FASTQ files generated from any NGS sequencing platform are converted into FASTA files. Artificially introduced 3′ adapter sequences are trimmed, and post-trimmed reads that are a minimum of 15 base pairs are filtered against contaminants. Reads that do not match to contaminants are screened for mature miRNA species (black box) which are further analyzed for statistical significance using mirnaTA. [file 2047-217X-3-20-S1.docx]

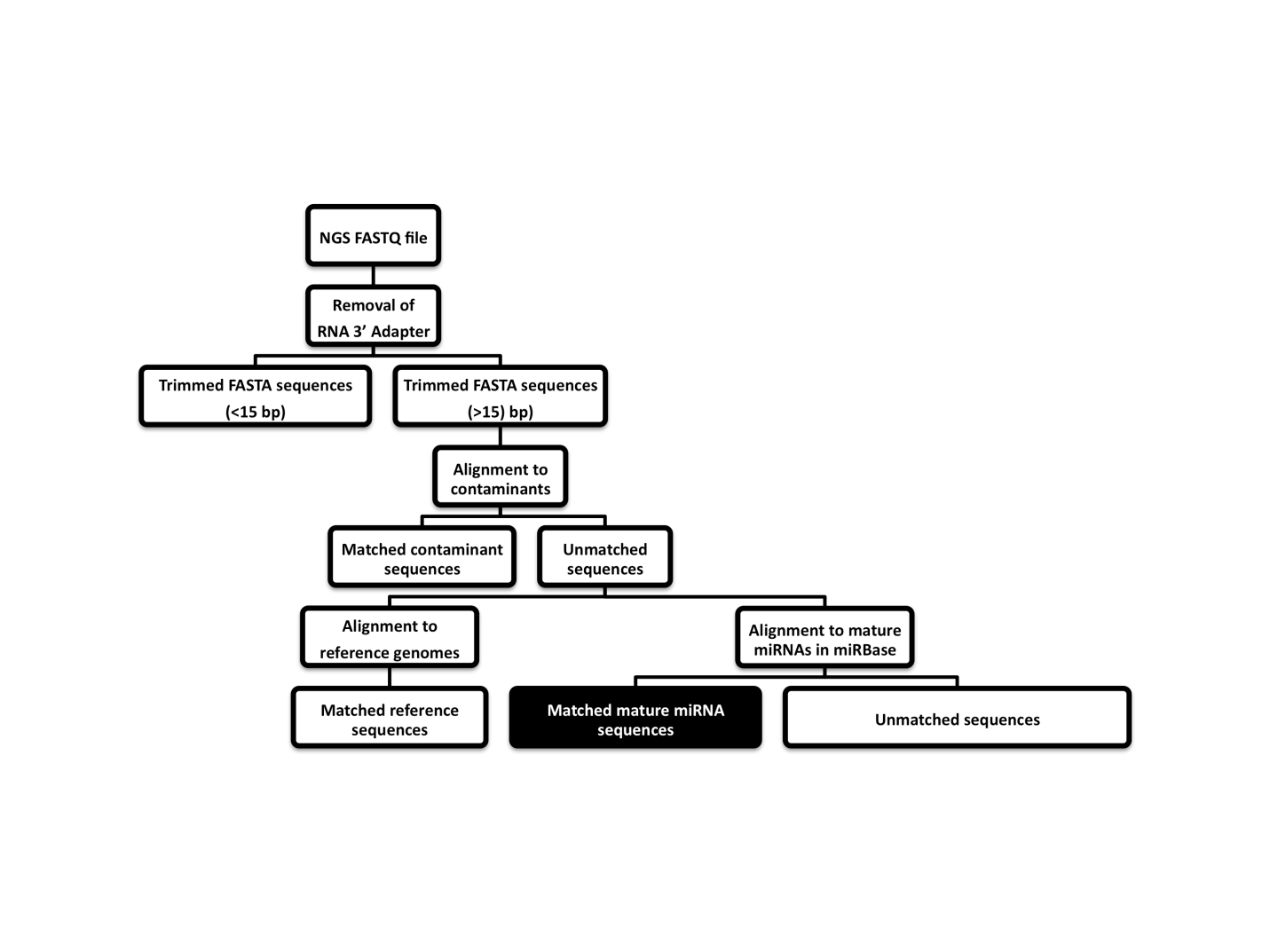


**Figure S1. Detailed steps for generating input files for mirnaTA.** FASTQ files generated from any NGS sequencing platform are converted into FASTA files. Artificially introduced 3’ adapter sequences are trimmed, and post-trimmed reads that are a minimum of 15 base pairs are filtered against contaminants. Reads that do not match to contaminants are screened for mature miRNA species (black box) which are further analyzed for statistical significance using mirnaTA.
